# Monitoring canopy SPAD based on UAV and multispectral imaging over fruit tree growth stages and species

**DOI:** 10.3389/fpls.2024.1435613

**Published:** 2024-08-01

**Authors:** Yirui Huang, Dongming Li, Xuan Liu, Zhenhui Ren

**Affiliations:** ^1^ Intelligent Sensor Network Engineering Research Center of Hebei Province, College of Information Engineering, Hebei GEO University, Shijiazhuang, China; ^2^ College of Mechanical and Electrical Engineering, Hebei Agricultural University, Baoding, China

**Keywords:** multispectral imaging, canopy SPAD value, vegetation index, reflection feature, texture feature

## Abstract

Chlorophyll monitoring is an important topic in phenotypic research. For fruit trees, chlorophyll content can reflect the real-time photosynthetic capacity, which is a great reference for nutrient status assessment. Traditional *in situ* estimation methods are labor- and time-consuming. Remote sensing spectral imagery has been widely applied in agricultural research. This study aims to explore a transferable model to estimate canopy SPAD across growth stages and tree species. Unmanned aerial vehicle (UAV) system was applied for multispectral images acquisition. The results showed that the univariate model yielded with Green Normalized Difference Vegetation Index (GNDVI) gave valuable prediction results, providing a simple and effective method for chlorophyll monitoring for single species. Reflection features (RF) and texture features (TF) were extracted for multivariate modeling. Gaussian Process Regression (GPR) models yielded better performance for mixed species research than other algorithm models, and the *R* ^2^ of the RF+TF+GPR model was approximately 0.7 in both single and mixed species. In addition, this method can also be used to predict canopy SPAD over various growth stages, especially in the third and fourth stages with *R* ^2^ higher than 0.6. This paper highlights the importance of using RF+TF for canopy feature expression and deep connection exploration between canopy features with GPR algorithm. This research provides a universal model for canopy SPAD inversion which can promote the growth status monitoring and management of fruit trees.

## 1 Introduction

Chlorophyll, as an important component of plant cells with the ability to absorb light and convert it into chemical energy, is critical to observe the photosynthetic capacity (Zarco-Tejada et al., 2016) and the nitrogen status of the fruit trees. Under the background of climate change and population growth, limited land resources and water resources make agricultural production face great pressure (Intergovermental Panel on Climate Change, 2022). Chlorophyll content monitoring in fruit trees can help predict yield and optimize plant varieties in biological research (Brewer et al., 2022), thereby increasing agricultural productivity and ensuring food security and sustainable development.

Traditionally, chlorophyll content was measured by chemical method (chromatographic separation), which is time- and labor-consuming. Spectral technology has gained widespread application in the non-destructive detection of chlorophyll content. The development of a portable spectrometer provides much convenience for field spectral collection for its mobility (Crocombe, 2018; Xiao et al., 2024). However, this approach is still labor-intensive and not suitable for large-scale area applications. With the evolvement of science and technology, remote sensing techniques have become popular gradually, promoting the development of high-throughput phenotypic research (Jafarbiglu and Pourreza, 2022). Satellite remote sensing technology helps us with large-scale data analysis. Nevertheless, for precision agriculture in small farms, satellite remote sensing is limited in spatial and temporal resolution. Unmanned aerial vehicle (UAV) remote sensing enables fast data acquisition with highly ground and temporal resolution; its advantages of simple operation and flexible application make it become an important means in agricultural monitoring research (Sankaran et al., 2019; Jafarbiglu and Pourreza, 2022).

Multispectral and hyperspectral sensors are imaging devices, which can simultaneously obtain two-dimensional spatial information and one-dimensional spectral information. The combination of spectral imaging and UAV can effectively facilitate data acquisition for a large area (Zhao et al., 2019). Lao et al. (2024) proposed a parameter/non-parameter combined model for canopy chlorophyll content retrieval of seven typical vegetation communities; high-spatial-resolution images were captured with UAV multispectral device. Wang et al. (2022) used UAV to estimate the percent green cover for high-throughput turfgrass; they found that multispectral images might offer a solution for non-green vegetation which is not captured by RGB images. Fu et al. used multi-platforms including analytical spectral device, UAV, and PlanetScope for water chlorophyll *a* concentration retrieval; the transfer learning methods were proposed for the ASD hyperspectral data to UAV and Planet platforms (Fu et al., 2023). Hyperspectral imagers have high spectral resolution and can detect more spectral information than multispectral cameras, but the processing method is more complicated for hyperspectral images (Hernanda et al., 2023). In addition, a multispectral camera is much cheaper than a hyperspectral camera (Zhang and Zhu, 2023), which is a significant factor considered in our research because economy is an important aspect that determines the possibility of application for a smallholder.

Vegetation index (VI) is calculated with reflectance of different spectral bands intending to select informative and sensitive spectral data and minimize non-informative information. It provides an important contribution to the assessment of plant status and successfully applied for chlorophyll estimation (Haboudane et al., 2008; Li et al., 2018), LAI monitoring (Hunt et al., 2010), and yield prediction (Zhou et al., 2017; Guan et al., 2019). However, previous research was mainly focused on plant evaluation for specific growth stage due to the great influence of phenological changes in biochemical content (Féret et al., 2017; Wang et al., 2018). Obviously, the growth model corresponding to different stages is inconvenient in the evaluation of the whole growing season. Therefore, it is necessary to build an applicable model throughout the growth cycle for chlorophyll retrieval. Moreover, Main et al. (2011) pointed out that for plant species (maize, cabbage, tomato, and several savanna tree species) with different degrees of chlorophyll content, indices using off-chlorophyll absorption center wavebands (690–730 nm) performed the most robust results. Similarly, in fruit tree studies, more research needs to be done to find common indices. More studies should continue to find a universal index for chlorophyll monitoring.

Texture feature, which refers to visual patterns or spatial arrangement of pixels, is another significant characteristic of images. It can provide spatial information on crop growth (Duan et al., 2019; Yogeshwari and Thailambal, 2021). In the rice aboveground biomass monitoring research, Xu et al. found that texture features can help to recognize the emergence of rice panicles, thus avoiding the overestimation at panicle initiation (Xu et al., 2022a). During the tillering stage to the booting stage, the texture features tended to be stable, which helps to the improvement of prediction accuracy (Xu et al., 2022b). Moreover, Li et al. got improved accuracy of nitrogen content models in winter wheat with the fusion of spectral and texture features (Li et al., 2023). Maimaitijiang et al. recognized that canopy texture features could offer canopy subtle structure characteristics which would be beneficial to soybean yield prediction (Maimaitijiang et al., 2020). However, there is a lack of literature on fruit trees about the potential of texture features (TF). Therefore, our research further explored the sensitivity of TF on the chlorophyll content evaluation of fruit trees (Abdelbaki and Udelhoven, 2022).

This study aims to assess the potential of vegetation indices (VIs), reflection features (RF), and TF in estimating the canopy SPAD values of fruit trees, combined with multiple machine learning algorithms. The emphasis focuses on inversion models that can be applied simultaneously to mixed tree species (apple tree and pear tree). The main contributions of this work are given as follows: (1) evaluate the VIs for canopy SPAD monitoring of single and mixed tree species with univariate algorithms, (2) compare the performance of the combined use of the RF and TF in estimating canopy SPAD based on deep learning algorithms, and (3) determine the optimal method for canopy SPAD inversion over various growth stages of mixed tree species.

## 2 Materials and methods

### 2.1 Study region

**Figure 1:**
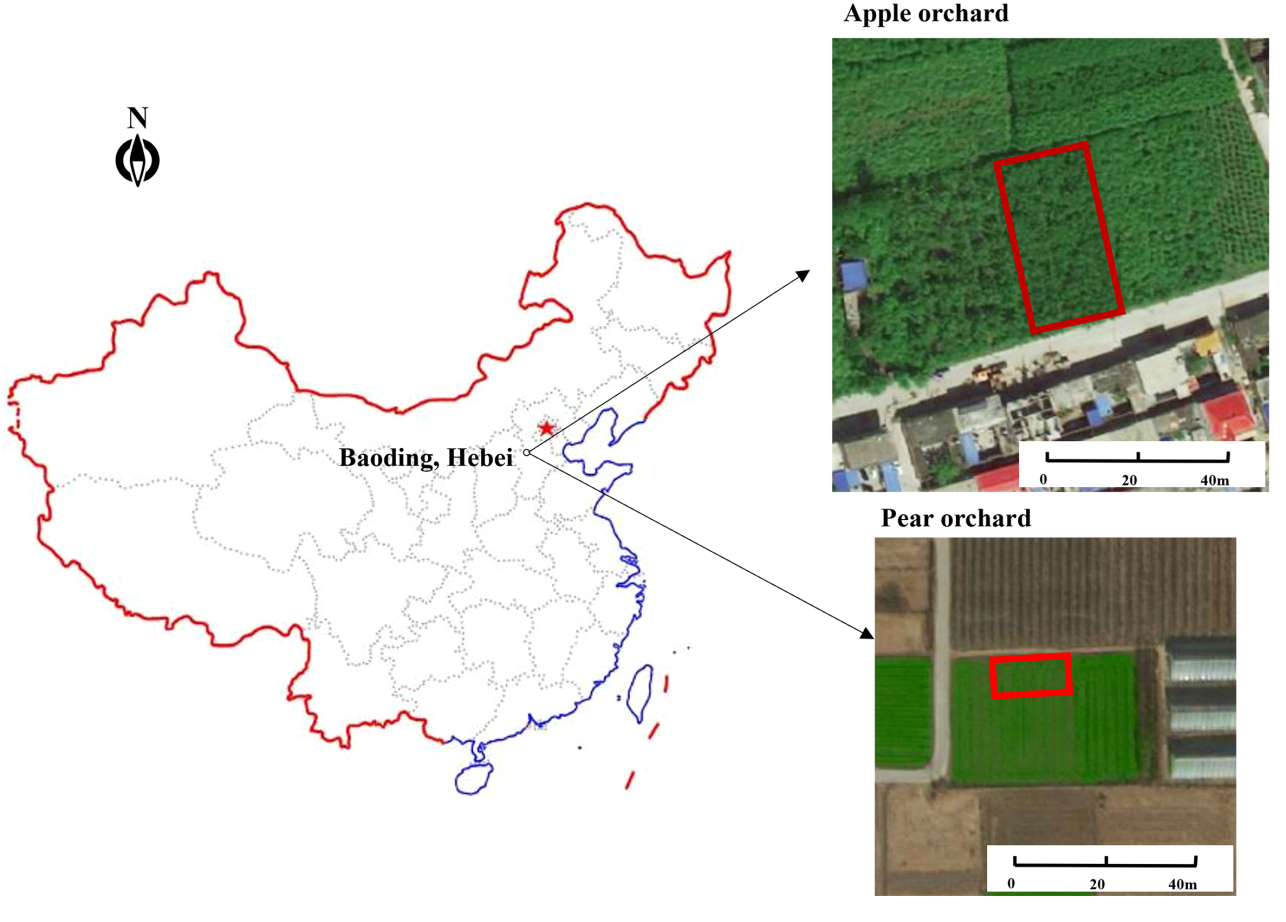
Location and satellite pictures of the experimental area.

This study was conducted in the city of Baoding, of Hebei province, China, with a temperate and monsoonal climate. The experimental field was an apple orchard (115.37° E, 38.90° N) with an elevation of approximately 25 m and a pear orchard (115.41° E, 38.84° N) with an elevation of approximately 30 m (**Figure 1**). The two orchards were watered by flowing irrigation, except when rain-fed. The total area size of the apple orchard is approximately 0.2 ha. A total of 91 “Morrissey apple” trees arranged in seven rows were planted in 2009. The apple trees were irrigated four times during the annual period, respectively—earlier during flowering and fruit growth beginning stage, the fruit growth and the volume increase stage, the time after the fruit picking stage, and later during the leaf senescence stage. The average apple tree height is 3.5 m. The pear orchard covers an area of approximately 0.27 ha, and a part of the whole orchard was set as research area, including 96 “Bergamot pear” trees arranged in eight rows and planted in 2015. The pear trees were irrigated four times during the annual period, especially in the time before the flowering stage, later of the flowering stage, the fruit growth and the volume increase stage, and the time after the fruit picking stage. The average pear tree height is 4.2 m. The experimental field was divided into microplots to obtain experimental data separately.

### 2.2 Field data collection

A chlorophyll meter is a portable device which allows the *in situ* quantification of total leaf chlorophyll by measuring optical densities at two separate wavebands, and the chlorophyll content can be estimated by SPAD values (Shu et al., 2021). In this study, the SPAD value was measured with a chlorophyll meter (TYS-4N Jinkelida Electronics, Beijing, China). The SPAD value was measured at six points and twice for each one; the average was taken as the final value. A comparative experiment between leaf chlorophyll content and SPAD value was carried out, and the results verified that the SPAD value was related to the chlorophyll content at the 0.01 level with a Pearson coefficient of more than 0.9 for both apple and pear tree leaves. For each microplot, the reference canopy SPAD is determined by averaging the SPAD values of 20 sample leaves which are healthy with an intact structure and located at the upper and different directions of the canopy.

**Figure 2:**
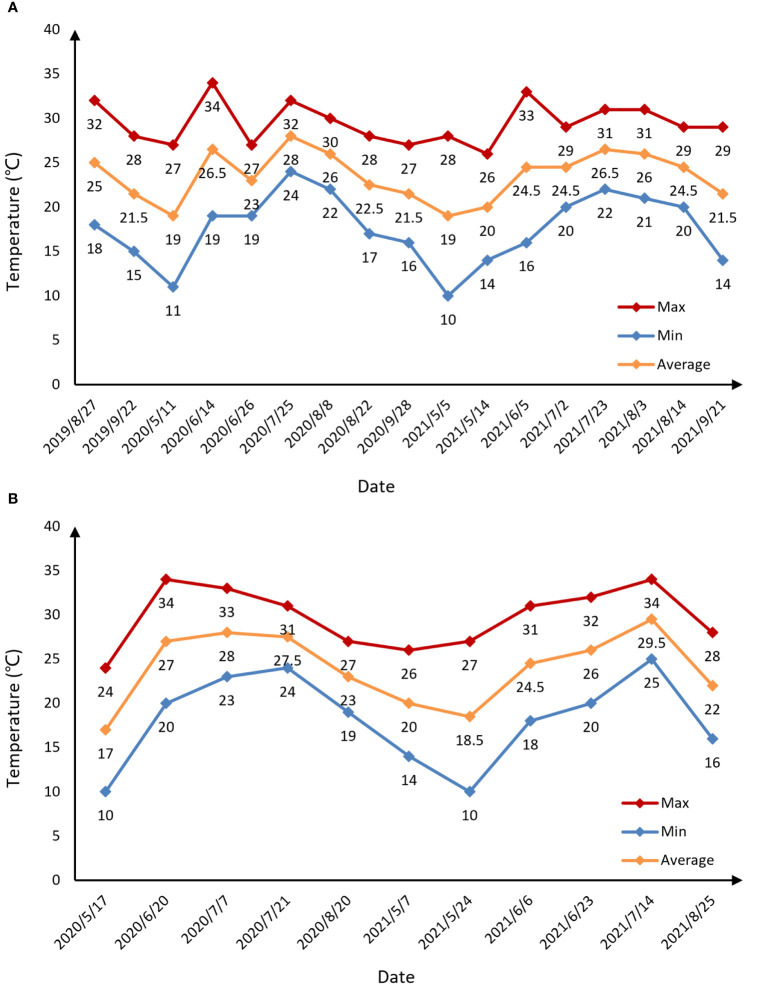
Temperature conditions of the experimental days in the apple orchard **(A)** and pear orchard **(B)**. “Max” represents the maximum temperature of the day. “Min” indicates the minimum temperature of the day. “Average” represents the average temperature of the day.

**Figure 3:**
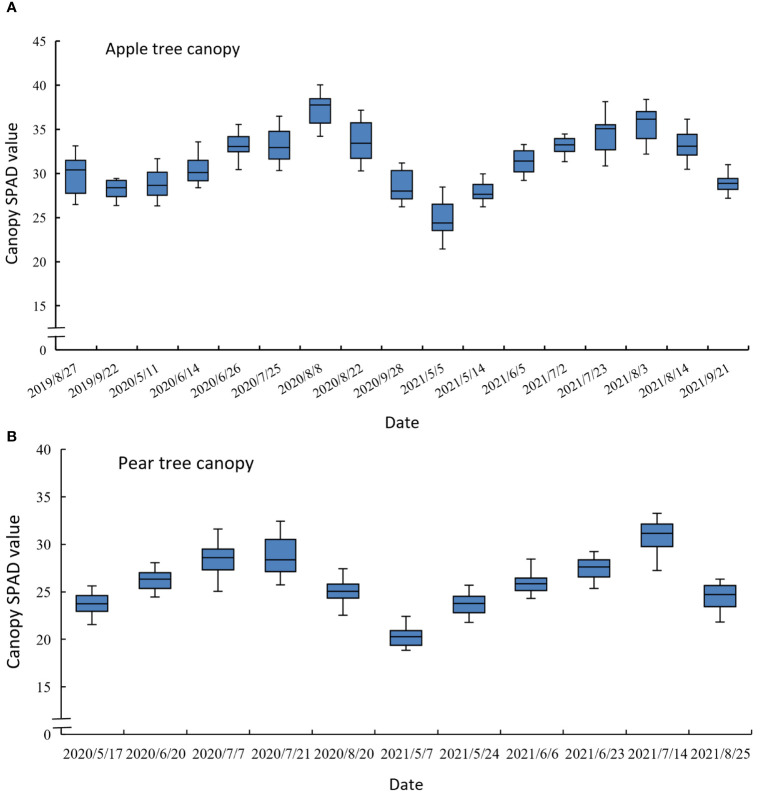
Statistical analysis map of canopy SPAD value in different growth stages of the apple tree **(A)** and the pear tree (**B**).

We implemented experiments from August 2019 to September 2021 in the apple orchard and from May 2020 to August 2021 in the pear orchard. The experimental date and corresponding growth stages are specified in **Table 1**. Each experimental day was a sunny day, and the temperature situation is shown in **Figure 2**. **Figure 3** describes the statistical analysis of the canopy SPAD value for fruit trees in different growth stages. It can be concluded that the canopy SPAD value increased from flowering until fruit maturation period and then began to decrease during the annual growth cycle. With the growth and development of tree leaves, the chlorophyll content in the leaves gradually increased, and when the fruits gradually matured, the chlorophyll content in the leaves began to decrease. In our study, the maturity time of pear trees was approximately 20 days earlier than that of apple trees.

**Table 1:** Schedule of experiments in the orchard.

	Experimental date		Growth stage	
For apple tree	2019 year	27 August	Fruit ripe for picking	BBCH 87
		22 September	Leaf senescence	───
	2020 year	11 May	Flowering and fruit growth beginning	BBCH 71
		14 June	Fruit growth and the volume increase	BBCH 72-73
		26 June		
		25 July	Fruit growth and ripening gradually	BBCH 73-87
		8 August		
		22 August	Fruit ripe for picking	BBCH 87
		28 September	Leaf senescence	───
	2021 year	5 May	Flowering and fruit growth beginning	BBCH 71
		14 May		
		5 June	Fruit growth and the volume increase	BBCH 72-73
		2 July	Fruit growth and ripening gradually	BBCH 73-87
		23 July		
		3 August		
		14 August	Fruit ripe for picking	BBCH 87
		21 September	Leaf senescence	───
For pear tree	2020 year	17 May	Flowering and fruit growth beginning	BBCH 71
		20 June	Fruit fall after flowering and size up	BBCH 71-73
		7 July	Fruit growth gradually	BBCH 73-87
		21 July	Fruit ripe for picking	BBCH 87
		20 August	Leaf senescence	───
	2021 year	7 May	Flowering and fruit growth beginning	BBCH 71
		24 May	Fruit fall after flowering and size up	BBCH 71-73
		6 June		
		23 June		
		14 July	Fruit ripe for picking	BBCH 87
		25 August	Leaf senescence	───

### 2.3 Multispectral image collection

**Figure 4:**
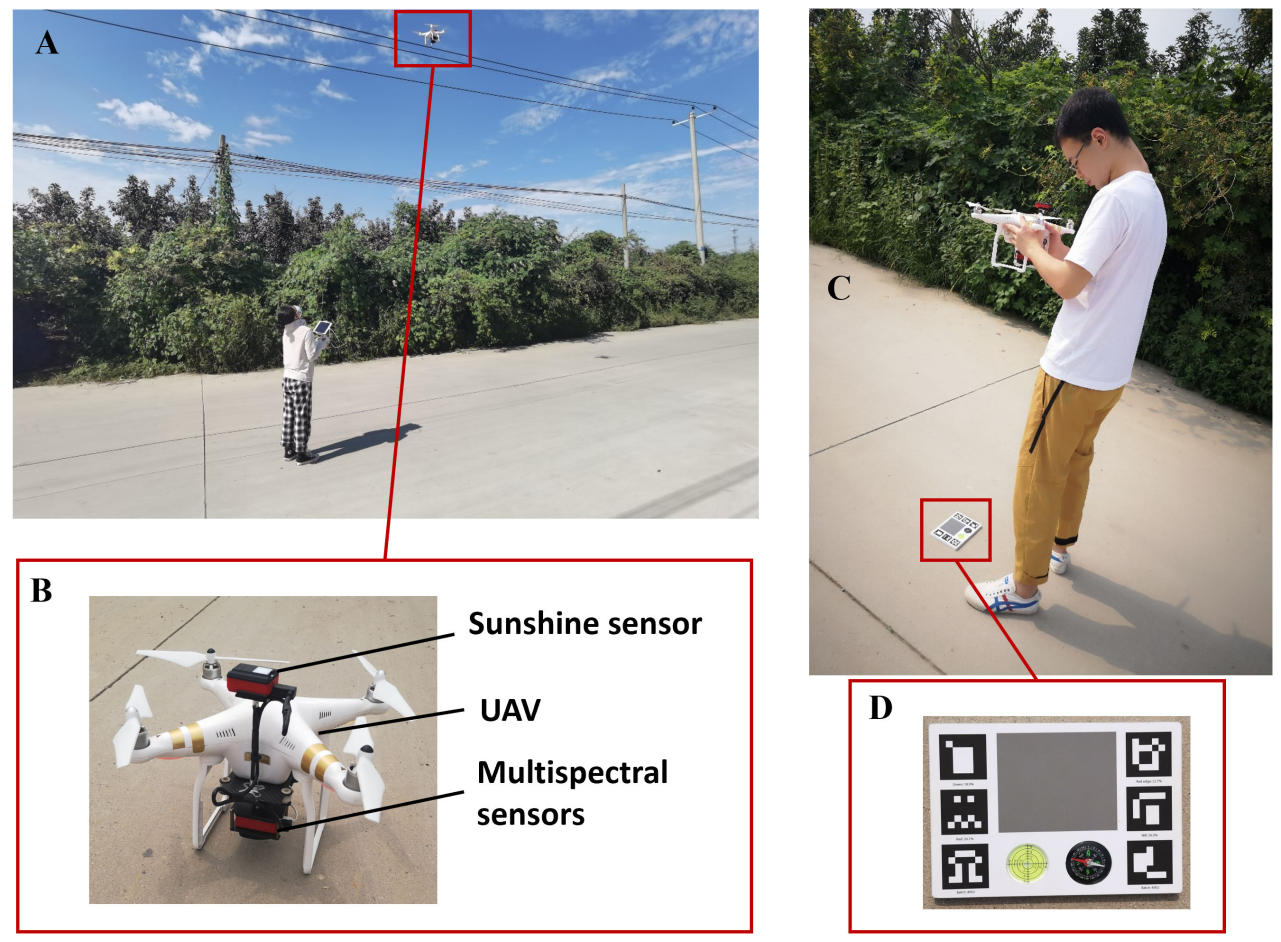
Multispectral images and radiometric calibration image collection. **(A)** Diagram of canopy image collection. **(B)** Parrot Sequoia camera and UAV equipment. **(C)** Diagram of radiometric calibration image collection. **(D)** Calibration reflectance panel.

Parrot Sequoia is a small and lightweight multispectral imager designed for agricultural remote sensing, with four spectral sensors and one RGB sensor (**Figure 4B**). The four spectral bands are situated in green (550 nm), red (660 nm), and near-infrared (790 nm) with bandwidth of 40 nm, respectively, and in red-edge (735 nm) with bandwidth of 10 nm. Furthermore, the imager was equipped with a sunshine sensor to compensate for the variability in sunlight conditions during different campaigns (**Figure 4A**) and to calibrate the images obtained with a calibration reflectance panel (**Figure 4D**) under different lighting conditions.

The UAV used in this study was DJI Phantom 3 Advanced (DJI Technology Co., Shenzhen, China), a low-cost quadrotor. Flight paths were generated in DJI GS Pro (https://www.dji.com/cn/ground-station-pro; DJI Technology Co., Shenzhen, China), ensuring images with 75% forward overlap and 85% side overlap. The drone flew autonomously along the flight path at a speed of 3.5 m/s, a height of 50 m above the ground level of the field. A total of 62 images were collected in the apple orchard, and 98 images were collected in the pear orchard under clear-sky conditions from 10:00 a.m. to 2:00 p.m. on each experiment day. The images have a spatial resolution of approximately 5 cm/pixel. In addition, two to three sets of calibration images were taken for reflectance correction to be applied (**Figure 4C**).

### 2.4 Data processing

**Figure 5:**
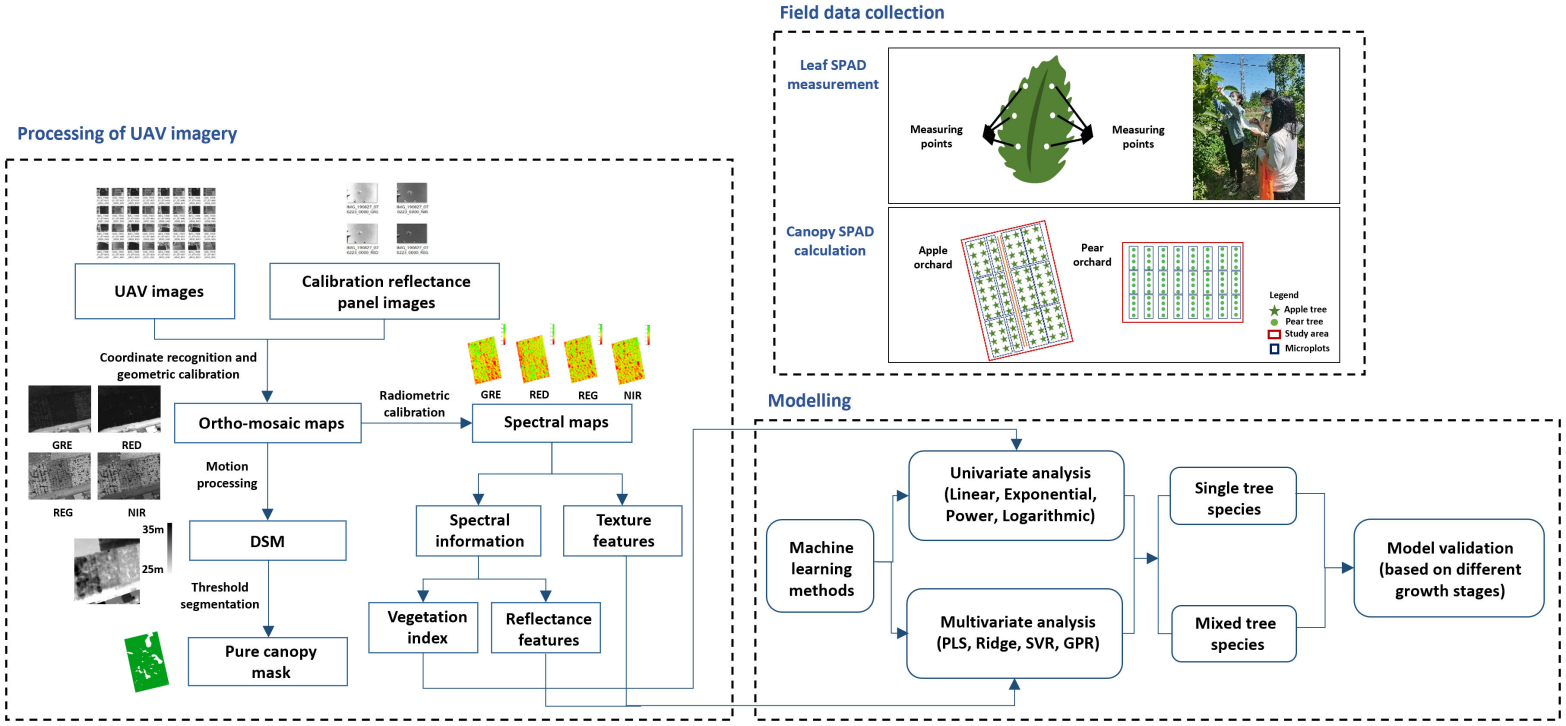
Workflow diagram of UAV imagery processing, field data collection, and modeling.

Machine learning methods were applied for canopy SPAD prediction modeling. The pre-processing of UAV imagery and the extraction of spectral information were computed with Pix4Dmapper software (https://www.pix4d.com.cn/pix4dmapper, Pix4D SA, Switzerland). The commonly used gray level co-occurrence matrix (GLCM) was selected to extract TF from multispectral UAV images. The TF were calculated using ENVI 5.3 software (https://envi.geoscene.cn, ITT Visual Information Solutions, USA), including mean, variance, homogeneity, contrast, dissimilarity, entropy, second moment, and correlation. **Figure 5** shows the workflow diagram of UAV imagery processing, field data collection, and modeling.

### 2.5 Univariate regression analysis method

Vegetation index (VI) is the combination of related reflectance and the integration of spectral data from two or more bands after a certain mathematical transformation (Gitelson et al., 2002). Narmilan et al. (2022) constructed different machine learning models with VIs for sugarcane chlorophyll content prediction. Madonsela et al. (2023) proved that the red-edge chlorophyll index and green chlorophyll index, using the red-edge variant centered at 705 nm, were the most useful for estimating maize LAI. In this study, we attempted to explore the utility of univariate methods (linear, exponential, power, and logarithmic regression) for tree canopy chlorophyll estimation with VI. The VIs used in our study for chlorophyll estimation are listed in **Table 2**. The original bands in the formulation of each VI was replaced by the closest available bands. The univariate regression analysis was implemented in Python environment through the “numpy” and “LinearRegression” packages.

**Table 2:** VIs selected from the literature.

VI name	VI formulation used in this study	References
NDVI (Normalized Difference Vegetation Index)	$\left(R_{NIR}-R_{RED}\right)/\left(R_{NIR}+R_{RED}\right)$	(Gitelson et al., 1996)
GNDVI (Green Normalized Difference Vegetation Index)	$\left(R_{NIR}-R_{GRE}\right)/\left(R_{NIR}+R_{GRE}\right)$	
REGNDVI (Red-edge Normalized Difference Vegetation Index)	$\left(R_{REG}-R_{GRE}\right)/\left(R_{REG}+R_{GRE}\right)$	
RVI (Ratio Vegetation Index)	$R_{NIR}/R_{RED}$	(Buschmann and Nagel, 1993; Gitelson and Merzlyak, 1994)
GRVI (Green Ratio Vegetation Index)	$R_{NIR}/R_{GRE}$	
REGRVI (Reg-edge Ratio Vegetation Index)	$R_{REG}/R_{GRE}$	
DVI (Difference Vegetation Index)	$R_{NIR}-R_{RED}$	(Tucker, 1979; Cao et al., 2013)
GDVI (Green Difference Vegetation Index)	$R_{NIR}-R_{GRE}$	
REGDVI (Red-Edge Difference Vegetation Index)	$R_{REG}-R_{GRE}$	
TVI (Triangular Vegetation Index)	$0.5\times \left[120\times \left(R_{NIR}-R_{GRE}\right)-200\times \left(R_{RED}-R_{GRE}\right)\right]$	(Broge and Leblanc, 2001; Haboudane et al., 2004, 2008)
MTVI (Modified Triangular Vegetation Index)	$\frac{1.5\times \left[1.2\times \left(R_{NIR}-R_{GRE}\right)-2.5\times \left(R_{RED}-R_{GRE}\right)\right]}{\sqrt{{\left(2\times R_{NIR}+1\right)}^{2}-\left(6\times R_{NIR}-5\times \sqrt{R_{NIR}}\right)-0.5}}$	
TCI (Triangular Chlorophyll Index)	$1.2\times \left(\frac{R_{REG}}{R_{GRE}}u\right)-1.5\times \left(\frac{R_{RED}}{R_{GRE}}\right)\times \sqrt{\left(\frac{R_{REG}}{R_{RED}}\right)}$	

### 2.6 Multivariate regression analysis method

Previous studies demonstrated that texture information can highlight the structure characteristics of plant and inhibit the saturation of models applied for plants with high heterogeneous features (Lu et al., 2018; Maimaitijiang et al., 2020). In our study, RF and TF were extracted from multispectral images and used for multivariate model inputs. The machine learning methods were linear and non-linear algorithms, as partial least square (PLS), ridge regression (Ridge), support vector regression (SVR), and gaussian process regression (GPR). All the models were trained in Python environment with JetBrains PyCharm (https://www.jetbrains.com/pycharm, JetBrains s.r.o., Prague, CZ).

PLS and Ridge methods were used for linear regression modeling. PLS method integrates the characteristics of principal component analysis, canonical correlation analysis, and multiple linear regression analysis, which has been used in bioinformatics, computer vision, and neuroinformatics (Mehmood and Ahmed, 2016). We implement the PLS model through the “PLSRegression” package; the parameter of “n_components” was optimized with “GridSearchCV”. Compared with general linear regression, regular terms (L2 norm penalty terms) (Dorugade and Kashid, 2010) are added in the objective function of Ridge models to solve the multicollinearity of independent variables and the overfitting in the training process (Ngo et al., 2003). The Ridge model was achieved with “sklearn.linear_model” package, the “alphas” was set as 0.5, and the “fit_intercept” was set as True.

SVR and GPR methods were used for non-linear regression modeling. Support vector machine (SVM) was used to distinguish the feature points with a hyperplane found by maximizing the interval (Dibike et al., 2001). SVR is the development and extension of SVM method (Ansari and Gholami, 2015). Radial basis function was set for the parameter of “kernel”. The parameters of “C” and “gamma” were optimized for loops. GPR is a non-parametric regression algorithm based on Bayesian theory (Wang et al., 2017). In the process of parameter adjustment, the posterior distribution on the objective function is defined, and the posterior distribution is constantly updated according to the predicted data until the posterior distribution basically fits the real distribution. GPR models were trained in Python, Matern was selected as “kernel” function, and the “nu” was set as 1.5.

### 2.7 Statistical analysis

The statistical analysis of original data is listed in **Table 3**. In single tree species research, the total data sets were divided into calibration set and validation set with the ratio of 3:1. In mixed tree species research, the calibration set included samples for apple, and pear trees were randomly selected from the previous calibration data sets in the same size (*n* = 150). The validation set was the same as the set in the single tree species studies. To explore the universality of the prediction models, all the canopy SPAD regression models were evaluated on the single tree species validation set of both apple and pear tree species.

**Table 3:** Statistical analysis of the canopy SPAD value.

Data set		Number of data	Max	Min	Mean	SD
Apple tree	All data	204	40.03	21.44	31.34	3.57
	Calibration set	153	40.03	21.44	31.32	3.65
	Validation set	51	39.53	24.42	31.40	3.35
Pear tree	All data	264	33.28	18.85	25.87	3.06
	Calibration set	198	33.28	18.85	25.83	3.12
	Validation set	66	31.62	19.28	26.01	2.90

In addition, prediction models were evaluated in terms of the coefficient of determination (*R* ^2^), the root mean square error (RMSE), and the relative root mean square error (RRMSE) (Sheng et al., 2020; Narmilan et al., 2022). A higher *R* ^2^ indicated that the model was more stable, and a lower RMSE or a lower RRMSE indicated great model accuracy. The Equations 1–3 display the formulas of *R^2^*, *RMSE* and *RRMSE*, respectively.


1 $$R^{2}=1-\frac{\sum_{i=1}^{n}{\left(Pred_{i}-Obs_{i}\right)}^{2}}{\sum_{i=1}^{n}{\left(\bar{Obs_{i}}-Obs_{i}\right)}^{2}}$$

2 $$RMSE=\sqrt{\frac{\sum_{i=1}^{n}{\left(Pred_{i}-Obs_{i}\right)}^{2}}{n}}$$

3 $$RRMSE=\frac{\sqrt{\frac{\sum_{i=1}^{n}{\left(Pred_{i}-Obs_{i}\right)}^{2}}{n}}}{Max\left(Obs_{i}\right)-Min\left(Obs_{i}\right)}$$

where $Pred_{i}$ are canopy SPAD values predicted with the regression models, $Obs_{i}$ are canopy SPAD value measurements in the field, $\bar{Obs_{i}}$ is the mean value of the $Obs_{i}$ , and n is the sample size. *R_c_* ^2^, RMSEC, and RRMSEC are performances of regression models trained on the calibration set, while *R_v_* ^2^, RMSEV, and RRMSEV are performances of regression models trained on validation set.

## 3 Results

### 3. 1 Canopy SPAD estimation with vegetation index

We evaluated the correlations between VI and canopy SPAD value with Pearson correlation coefficient (*R*) (**Table 4**). The results showed that GNDVI had the highest correlation with canopy SPAD value with a Pearson coefficient of 0.753 for apple tree and 0.718 for pear tree; GRVI had the second highest Pearson coefficient of 0.646 for apple tree and 0.714 for pear tree. It is worth noting that there are some indicators that are not correlated at the *p* level.

**Table 4:** Significance test between VI and the canopy SPAD value.

I	NDVI	GNDVI	REGNDVI
R (apple/pear tree)	0.483^∗∗^/0.601^∗∗^	**0.753^∗∗^/0.718^∗∗^**	0.549^∗∗^/**0.536^∗∗^**
VI	RVI	GRVI	REGRVI
R (apple/pear tree)	0.235^∗∗^/0.613^∗∗^	**0.646^∗∗^/0.714^∗∗^**	**0.623^∗∗^**/0.403^∗∗^
VI	DVI	GDVI	REGDVI
R (apple/pear tree)	ns /0.324^∗∗^	0.057/0.488^∗∗^	−0.027/0.381^∗∗^
VI	TVI	MTVI	TCI
R (apple/pear tree)	−0.040/0.248^∗∗^	0.433^∗∗^/0.382^∗∗^	0.545^∗∗^/0.396^∗∗^

∗∗, significance at p < 0.01; ns, no significance. Bold values: The vegetation index corresponding to the bold values has higher model performances.

**Figure 6:**
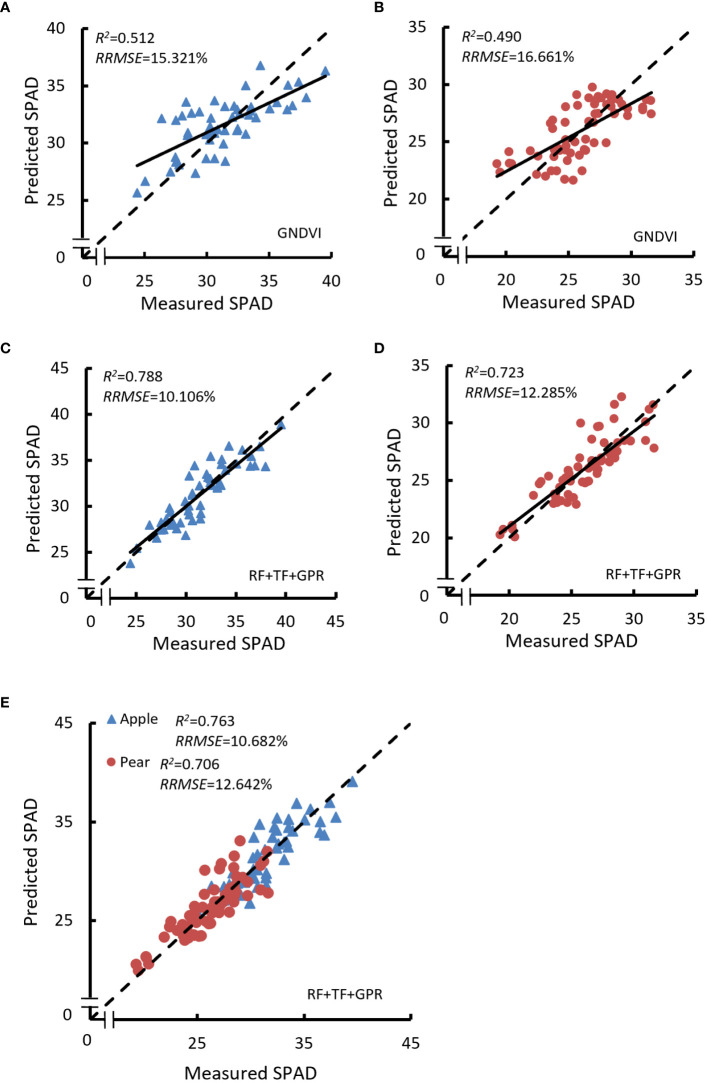
Relationship between the predicted and measured SPAD value of canopy SPAD. **(A)** Calculated with GNDVI in single tree species research for apple tree. **(B)** Yielded with GNDVI in single tree species research for apple tree. **(C)** Obtained with RF+TF+GPR in single tree species research for apple tree. **(D)** Trained with RF+TF+GPR in single tree species research for apple tree. **(E)** Done with RF+TF+GPR in mixed tree species research.

In single tree species research, we conducted prediction models with VIs correlated with canopy SPAD values. The model results with higher prediction accuracy on the validation set are shown in **Table 5**. Considering the model results in both apple tree and pear tree, GNDVI was taken as the most potential VI for canopy SPAD evaluation by a simple monistic model, and *R* ^2^ was 0.512 and 0.490 on the validation set for apple trees (**Figure 6A**) and pear trees (**Figure 6B**), respectively.

**Table 5:** Performance of the models yielded in single tree species research.

Tree species	Model type		Calibration			Validation		
			*R_c_* ^2^	RMSEC	RRMSEC	*R_v_* ^2^	RMSEV	RRMSEV
For apple tree	GNDVI		0.583	2.350	12.641%	0.512	2.315	15.321%
	REGRVI		0.396	2.827	15.207%	0.359	2.653	17.558%
	GRVI		0.431	2.743	14.755%	0.338	2.697	17.849%
	RF	PLS	0.598	2.305	12.399%	0.519	2.300	15.222%
		Ridge	0.598	2.306	12.405%	0.518	2.302	15.235%
		SVR	0.634	2.201	11.840%	0.585	2.135	14.130%
		GPR	0.883	1.242	6.681%	0.700	1.815	12.012%
	RF + TF	PLS	0.806	1.601	8.612%	0.707	1.795	11.880%
		Ridge	0.832	1.490	8.015%	0.703	1.807	11.960%
		SVR	0.616	2.254	12.125%	0.489	2.368	15.672%
		GPR	0.928	0.975	5.245%	0.788	1.527	10.106%
For pear tree	GNDVI		0.515	2.169	15.031%	0.490	2.056	16.661%
	GRVI		0.524	2.147	14.879%	0.483	2.070	16.775%
	REGNDVI		0.494	2.216	15.357%	0.495	2.045	16.572%
	RF	PLS	0.614	1.934	13.403%	0.550	1.932	15.656%
		Ridge	0.612	1.938	13.430%	0.554	1.921	15.567%
		SVR	0.837	1.257	8.711%	0.733	1.486	12.042%
		GPR	0.834	1.268	8.787%	0.721	1.521	12.326%
	RF + TF	PLS	0.828	1.292	8.954%	0.737	1.475	11.953%
		Ridge	0.828	1.290	8.940%	0.739	1.469	11.904%
		SVR	0.738	1.595	11.053%	0.489	2.057	16.669%
		GPR	0.878	1.089	7.547%	0.723	1.516	12.285%

In the mixed tree species research, canopy SPAD estimation models were yielded by using GNDVI and GRVI (which were the common VIs in the first three optimal models in single tree species research); the evaluation parameters of the models are listed in **Table 6**. However, it should be noted that the prediction accuracy of this method was not satisfactory. The model *R* ^2^ was 0.2–0.35, which was not reliable enough for canopy SPAD retrieval.

**Table 6:** Performance of the models as yielded in mixed tree species research.

Model type		Calibration			Validation (apple tree)			Validation (pear tree)		
		*R_c_* ^2^	RMSEC	RRMSEC	*R_v_* ^2^	RMSEV	RRMSEV	*R_v_* ^2^	RMSEV	RRMSEV
GNDVI		0.566	2.870	13.551%	0.221	2.926	19.365%	0.341	2.337	18.938%
GRVI		0.578	2.830	13.362%	0.262	2.847	18.842%	0.264	2.470	20.016%
RF	PLS	0.454	3.219	15.198%	0.039	3.249	21.502%	0.300	2.412	19.546%
	Ridge	0.516	3.045	14.377%	0.060	3.214	21.271%	0.421	2.190	17.747%
	SVR	0.815	1.874	8.848%	0.556	2.208	14.613%	0.687	1.609	13.039%
	GPR	0.870	1.573	7.427%	0.681	1.873	12.400%	0.707	1.559	12.634%
RF+TF	PLS	0.546	2.936	13.862%	0.253	2.864	18.954%	0.123	2.695	21.840%
	Ridge	0.749	2.181	10.297%	0.476	2.398	15.870%	0.614	1.789	14.498%
	SVR	0.927	1.178	5.562%	0.566	2.183	14.447%	0.322	2.369	19.198%
	GPR	0.943	1.040	4.910%	0.763	1.614	10.682%	0.706	1.560	12.642%

### 3.2 Canopy SPAD estimation with image features

**Figure 7:**
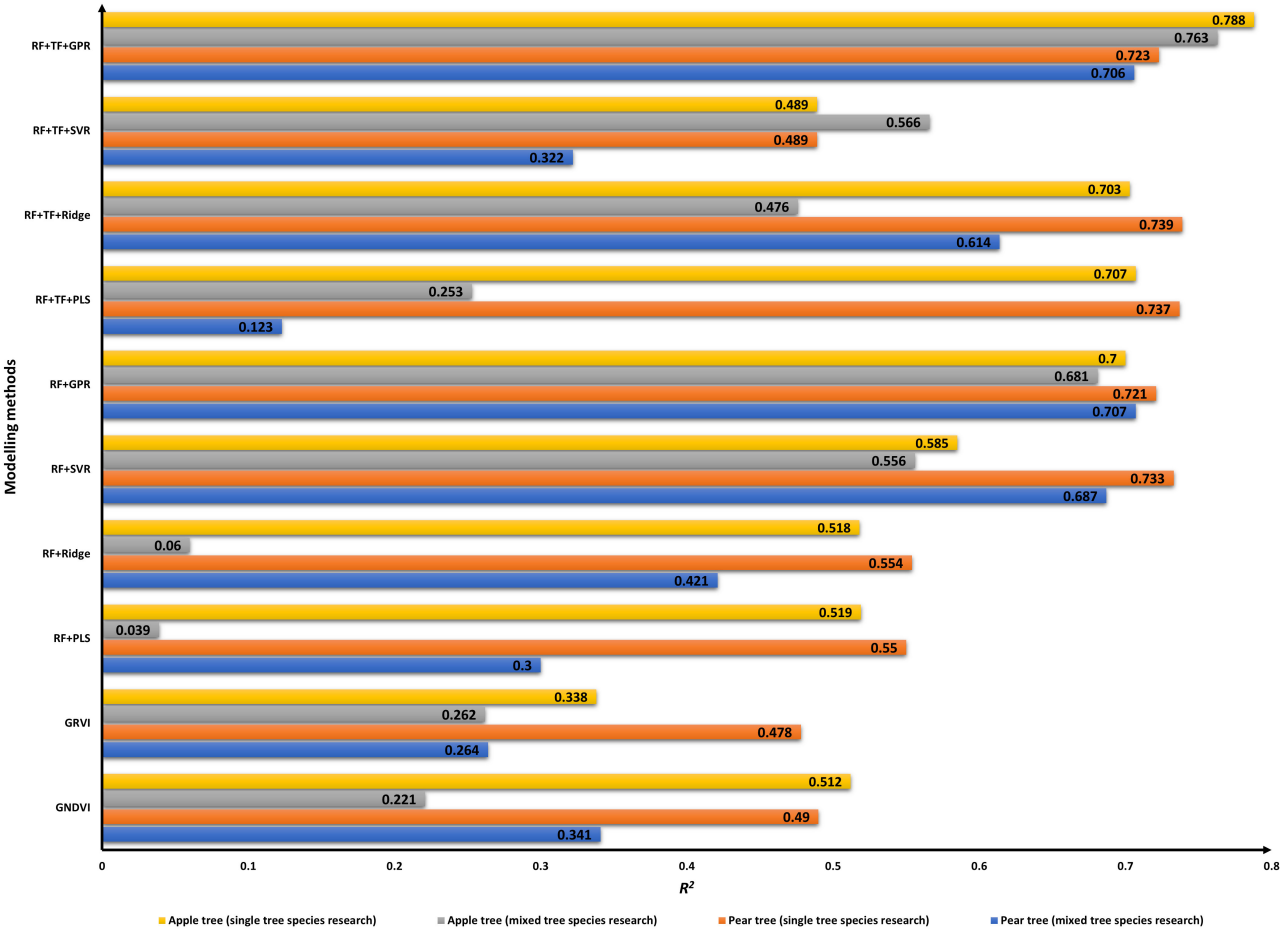
Model performance for canopy SPAD estimation.

Multivariate models were established with image features by using PLS, Ridge, SVR, and GPR algorithms; the relevant results are shown in **Table 5** and **Table 6**. For an intuitive comparison of model performance, the *R* ^2^ value on the validation data of the models is displayed in **Figure 7** (the univariate model results with GNDVI and GRVI were also listed to make a comprehensive comparison between modeling methods).

In single tree species research, prediction models were implemented with RF firstly. For apple trees, PLS and Ridge were performed equally; the *R_v_* ^2^ was approximately 0.52, and the GPR model yielded better results with *R_v_* ^2^ of 0.700 than the SVR model with *R_v_* ^2^ of 0.585. For pear trees, PLS and Ridge were also performed equally with *R_v_* ^2^ of 0.55. The *R_v_* ^2^ of the SVR model was 0.733, and RRMSEV was 12.042%, which were a little superior to the results of the GPR model (*R_v_* ^2 ^= 0.721, RRMSEV = 12.326%). In the latter research, the fusion of RF and TF was set as model inputs; the prediction accuracy of the models obtained by the same algorithm was improved, except by the SVR method. For apple trees, *R_v_* ^2^ of the PLS and Ridge models were up to 0.70, and *R_v_* ^2^ of the GPR model was up to 0.788 (**Figure 6C**). For pear trees, *R_v_* ^2^ of the PLS and Ridge models were up to 0.73, and *R_v_^2^* of the GPR model was slightly improved to 0.723 (**Figure 6D**).

In mixed tree species research, when RF were selected as model inputs, the validation performance of the PLS and Ridge models was not reliable, especially for apple trees; *R_v_* ^2^ was less than 0.1. The GPR model got the best accuracy with *R_v_* ^2^ of 0.681 for apple trees and 0.707 for pear trees. When RF and TF were mixed for model variables, the validation performance of the PLS and Ridge models were obviously improved, and the Ridge model yielded *R_v_* ^2 ^= 0.476 for apple trees and *R_v_* ^2 ^= 0.614 for pear trees. The GPR model is also the best prediction model with *R_v_* ^2^ of 0.763 for apple trees and 0.706 for pear trees (**Figure 6E**).

### 3.3 Canopy SPAD estimation over the various growth stages

**Figure 8:**
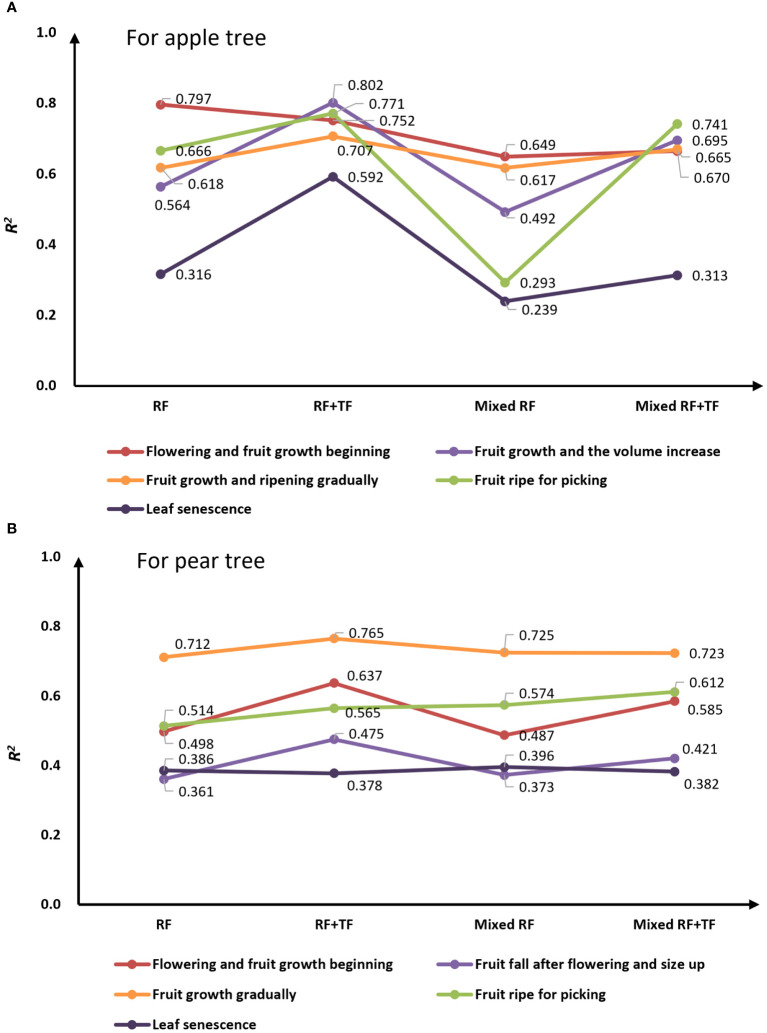
Prediction accuracy for the various growth stages of the apple tree **(A)** and the pear tree **(B)**.

To evaluate the growth periods’ prediction performance of the GPR method, the canopy SPAD prediction was carried out at different stages in 2021. **Figure 8** shows the accuracy of the models trained with various types of inputs by *R* ^2^ metrics over the various growth stages.

In the single tree species inversion, for apple trees, the prediction model yielded well accuracy with only RF during the first stages (flowering and fruit growth beginning) with *R* ^2^ of 0.797. In the latter four growth stages, the GPR models got a higher *R* ^2^ value with RF+TF than with only RF. It was 0.592 during leaf senescence stage and approximately 0.7 to 0.8 in the remaining three stages. For pear trees, the GPR models yielded better results with RF+TR than with only RF during the first four stages; the *R* ^2^ value was approximately 0.475–0.765, and the highest accuracy was obtained in the fruit-growth-gradually stage with *R* ^2 ^= 0.765.

In the mixed tree species inversion, for apple trees, the prediction was that the *R* ^2^ values obtained with RF+TF were all greater than those obtained with only RF, which especially increased from 0.492 to 0.695 in the second growth stage and increased from 0.293 to 0.741 in the fourth growth stage. For pear trees, the model results trained with RF+TF got the highest *R* ^2^ value of 0.723 in the third growth stages and got an *R* ^2^ value between 0.382 and 0.612 in the remaining four stages.

## 4 Discussion

### 4.1 Vegetation index for canopy SPAD inversion

Vegetation index has been widely used in agricultural production research, including disease monitoring, biomass detection, etc. The GNDVI was originally developed for chlorophyll concentration measurement in maize leaves (Daughtry et al., 2000). Hunt et al. found that GNDVI was highly relevant with leaf area index and nitrogen status for winter wheat (Hunt et al., 2010). Shanahan et al. found that GNDVI was of great value in predicting corn grain yield (Shanahan et al., 2001). In this paper, prediction models trained with GNDVI yielded an *R_v_* ^2^ value of 0.512 for apple trees and 0.490 for pear trees in single tree species research. However, the results in mixed tree species research showed that GDVI was not a reliable index for canopy SPAD inversion. In the past studies of chlorophyll retrieval using VIs, researchers found that the sensitivity of vegetation index was greatly affected by the density of the crop canopy (Yang et al., 2022). In our study, apple trees are much older than pear trees, and they have many differences in canopy structure, specifically in canopy volume and canopy density. Therefore, when GNDVI was used for chlorophyll assessment in single tree species, the model yielded relatively reliable results. However, the sensitivity of GNDVI to chlorophyll retrieval decreased under the interference of canopy structure heterogeneity in the research of mixed tree species.

### 4.2 Comparison of modeling methods

Comparing the models’ results, the prediction accuracies of the PLS and Ridge models were close, except that the Ridge model performed better than the PLS model with RF+TF in mixed tree species research. However, since that is not a universal phenomenon, the Ridge method cannot be considered superior to the PLS method. SVR models did not do well with the huge distance between the calibration and validation performances, especially in mixed tree species studies. The main factor that caused poor performance may be the several structural parameters, which should be continuously adjusted in model training. Inappropriate parameters would greatly impact prediction performance. From the results of the model based on spectral features or the combination of spectral and texture features, we can see that the SVR model yielded robust results when having relatively few variables. It is worth noting that the GPR model has excellent performance in modeling as shown in **Figure 7**, yielding reliable prediction accuracies on the validation set with the different combinations of variables. In contrast to the non-linear model, the linear model has a weaker ability to explore the deep connections between input features. The results indicated that the more information covered by the inputs contributes to the higher accuracy for linear model, which is in line with the study of Maimaitijiang et al. (2020).

### 4.3 Contribution of texture features in canopy SPAD estimation

In our study, we focus on the contribution of TF to the canopy SPAD evaluation over tree species. In single tree species research, compared with the model performances yielded by RF+TF of which were obtained by only RF, the evaluation parameters were generally improved. For apple tree, the linear PLS model had *R* ^2^ increased by 36.2%, and the non-linear GPR model had *R* ^2^ increased by 12.6%. For pear tree, the *R* ^2^ of the linear Ridge model increased from 0.554 to 0.739, an increase of 33.4%. For the non-linear GPR model of pear tree, *R* ^2^ had increased by 0.3%. In the mixed tree species research, TF have significant optimization ability for linear Ridge models; the validation *R* ^2^ increased from 0.06 to 0.476 for apple tree and from 0.421 to 0.614 for pear tree. In the GPR model results, the validation *R* ^2^ increased from 0.681 to 0.763 for apple tree. Although the improvement of the model results for pear tree was slightly smaller than that of apple tree, the importance of texture features in canopy SPAD monitoring research should not be ignored.

For the same tree species, the model results obtained with mixed tree species information were a little weaker than the results obtained with single tree species information, but the reduction was within the expectation considering the effect of tree heterogeneity. Chlorophyll content can be effectively monitored with tree canopy RF, and TF can facilitate the extraction of diversity among tree species, which is beneficial to yield a chlorophyll content assessment model with higher accuracy and more universality ability. In the results obtained by Li et al. (2018) for remote sensing imaging-based canopy chlorophyll estimation, the SVR model with modified NDVI yielded *R* ^2^ = 0.667 on the validation set. In this paper, it can be concluded that canopy SPAD would be monitored with the RF+TF+GPR method, *R* ^2^ on the validation set in single tree species was 0.788 for apple tree and 0.723 for pear tree, and *R* ^2^ on the validation set over different tree species was 0.763 and 0.706 for apple tree and pear tree, respectively. The results go beyond a previous report, successfully getting a higher prediction accuracy on original reflectance and texture data. Nevertheless, it is worth noting that apple trees and pear trees both belong to the wild fruit *Rosaceae* plant resources, which might be one of the reasons supporting the high prediction accuracy in the mixed species study. More research should be carried out to explore the significance of RF+TF for other species with greater canopy differences. Moreover, compared with satellite remote sensing technique, UAV imagery has a wider range of application circumstance as high-throughput phenotyping method, and the UAV images have a higher special resolution which is beneficial to the extraction of TF. These advantages of UAV imagery are conducive to the popularization and application in agriculture.

From the overall trend of the lines in **Figure 8**, it was noted that the optimization ability of texture features to model prediction accuracy is more prominent for apple orchards than that for pear orchards. Considering the actual planting situation, the high canopy coverage of the apple orchard leads to serious airtight phenomenon and poor light transmittance, resulting in invalid leaves exiting in the canopy which get insufficient illumination. Being different from the apple orchard, the tree rows spacing is regular in the pear orchard, and trees grow in uniform and adequate light conditions. Therefore, the contribution of TF for canopy SPAD inversion for pear orchard was not as prominent as that for the apple orchard. However, the study results of two different tree species indicated that the contribution of TF was still significant.

### 4.4 Analysis of model performance in different growth stages

By analyzing the model performance obtained with RF+TF in mixed tree species research, it can be found that the models yielded high prediction accuracy during flowering stage to picking stage and the *R* ^2^ values between 0.66 and 0.75 for apple trees as well as the *R^2^* values between 0.58 and 0.73 for pear trees, except at the second stage. This may be attributed to the natural falling of fruits and leaves in the second stage for pear trees. As the leaves fall off, the canopy density will be reduced, and the background effect of canopy gaps will cause the inhomogeneity of the reflectivity scene (Brewer et al., 2022). Hence, the optimal conditions for predicting chlorophyll content are missing in this stage. In addition, the prediction accuracy of the chlorophyll model at the leaf senescence stage was less than 0.4 for both apple and pear trees due to the low chlorophyll content. Leaf senescence is a degenerative process; chlorophyll and its associated proteins break down during this stage, resulting in most bands being absorbed (Guo et al., 2021; Brewer et al., 2022). In total, the RF+TF+GPR model can effectively monitor the canopy SPAD for both apple and pear orchards at different growth stages. More experiment area and tree species need to be developed to improve the model universality and prediction accuracy.

**Figure 9:**
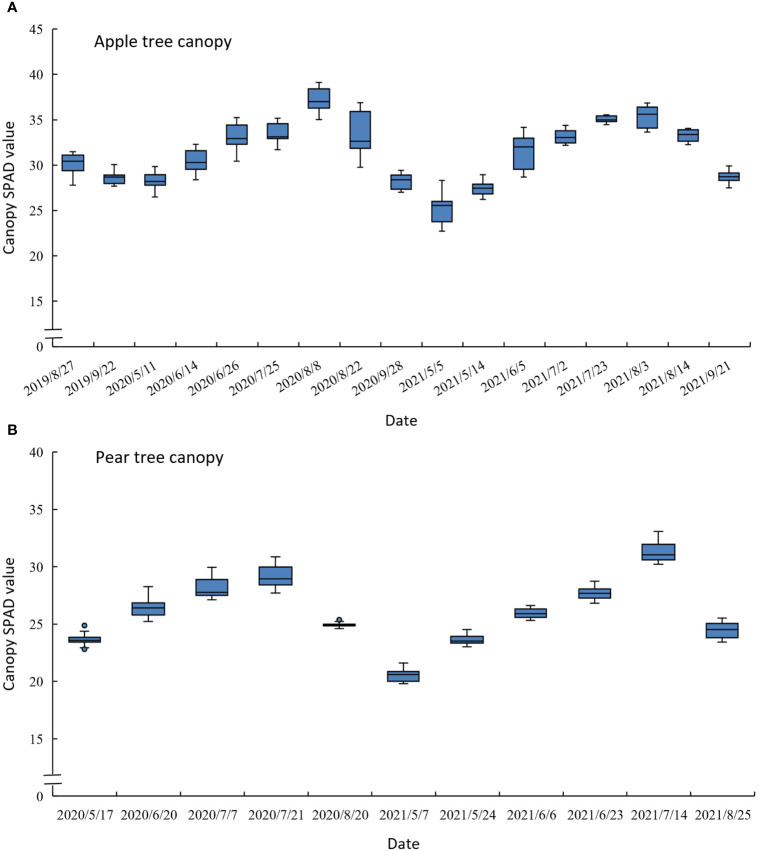
Statistical analysis map of the predicted canopy SPAD value (using GPR+RF+TF mixed tree species model) in different growth stages of the apple tree **(A)** and the pear tree (**B**).

In addition, **Figure 9** displays the statistical analysis results of the predicted canopy SPAD at different growth stages by using the mixed tree species model yielded by the GPR+RF+TF method. Compared with **Figure 3**, which is the analysis results of the measured canopy SPAD value, the predicted results can effectively respond to the change in trend of the canopy chlorophyll content of fruit trees over the various growth periods.

## 5 Conclusion

In this research, we presented the canopy SPAD evaluation in apple and pear orchards using UAV multispectral imagery. For univariate models, GNDVI was an efficient index in single tree species research, but the same conclusion cannot be supported in mixed tree species research. For multivariate models, GPR algorithm performed better than other machine learning methods; the RF+TF+GPR model yielded *R* ^2^ value of 0.788 for apple trees and 0.723 for pear trees in single tree species research and trained *R* ^2^ value of 0.763 for apple trees and 0.706 for pear trees in mixed tree species research. Compared with the RF+GPR model results, TF can retrieve more canopy structure information, promoting the prediction accuracy of canopy SPAD. Moreover, the RF+TF+GPR method is suitable for canopy SPAD during the growth-beginning stage to the ripe-for-picking stage, which is beneficial for canopy SPAD monitoring over various stages. The future scope of this work would focus on more fruit tree areas and species using large amounts of data to improve the universality and accuracy of the models.

## Data availability statement

The raw data supporting the conclusions of this article will be made available by the authors, without undue reservation.

## Author contributions

YH: Conceptualization, Data curation, Methodology, Supervision, Writing – original draft, Writing – review & editing. DL: Conceptualization, Data curation, Writing – original draft, Writing – review & editing. XL: Methodology, Validation, Writing – original draft, Writing – review & editing. ZR: Funding acquisition, Supervision, Writing – original draft, Writing – review & editing.

## References

[B1] Abdelbaki A. Udelhoven T. (2022). A review of hybrid approaches for quantitative assessment of crop traits using optical remote sensing: research trends and future directions. Remote Sens.-Basel. 14, 3515. doi: 10.3390/rs14153515

[B2] Ansari H. R. Gholami A. (2015). An improved support vector regression model for estimation of saturation pressure of crude oils. Fluid Phase Equilibr. 402, 124–132. doi: 10.1016/j.fluid.2015.05.037

[B3] Brewer K. Clulow A. Sibanda M. Gokool S. Naiken V. Mabhaudhi T. (2022). Predicting the chlorophyll content of maize over phenotyping as a proxy for crop health in smallholder farming systems. Remote Sens. -Basel. 14, 518. doi: 10.3390/rs14030518

[B4] Broge N. H. Leblanc E. (2001). Comparing prediction power and stability of broadband and hyperspectral vegetation indices for estimation of green leaf area index and canopy chlorophyll density. Remote Sens. Environ. 76, 156–172. doi: 10.1016/S0034-4257(00)00197-8

[B5] Buschmann C. Nagel E. (1993). *In vivo* spectroscopy and internal optics of leaves as basis for remote sensing of vegetation. Int. J. Remote Sens. 14, 711–722. doi: 10.1080/01431169308904370

[B6] Cao Q. Miao Y. Wang H. Huang S. Cheng S. Khosla R.. (2013). Non-destructive estimation of rice plant nitrogen status with Crop Circle multispectral active canopy sensor. Field Crop Res. 154, 133–144. doi: 10.1016/j.fcr.2013.08.005

[B7] Crocombe R. A. (2018). Portable spectroscopy. Appl. Spectrosc. 72, 1701–1751. doi: 10.1177/0003702818809719 30335465

[B8] Daughtry C. S. T. Walthall C. L. Kim M. S. de Colstoun E. B. Mcmurtrey J. E. (2000). Estimating corn leaf chlorophyll concentration from leaf and canopy reflectance. Remote Sens. Environ. 74, 229–239. doi: 10.1016/S0034-4257(00)00113-9

[B9] Dibike Y. Velickov S. Solomatine D. Abbott M. (2001). Model induction with support vector machines: Introduction and applications. J. Comput. Civil Eng. 15, 208–216. doi: 10.1061/(ASCE)0887-3801(2001)15:3(208)

[B10] Dorugade A. V. Kashid D. N. (2010). Variable selection in linear regression based on ridge estimator. J. Stat. Comput. Sim. 80, 1211–1224. doi: 10.1080/00949650903012413

[B11] Duan B. Liu Y. Gong Y. Peng Y. Wu X. Zhu R.. (2019). Remote estimation of rice LAI based on Fourier spectrum texture from UAV image. Plant Methods 15, 124. doi: 10.1186/s13007-019-0507-8 31695729 PMC6824110

[B12] Féret J. B. Gitelson A. A. Noble S. D. Jacquemoud S. (2017). PROSPECT-D: Towards modeling leaf optical properties through a complete lifecycle. Remote Sens. Environ. 193, 204–215. doi: 10.1016/j.rse.2017.03.004

[B13] Fu B. Li S. Lao Z. Yuan B. Liang Y. He W. (2023). Multi-sensor and multi-platform retrieval of water chlorophyll a concentration in karst wetlands using transfer learning frameworks with ASD, UAV, and Planet CubeSate reflectance data. Sci. Total Environ 901, 165963. doi: 10.1016/j.scitotenv.2023.165963 37543316

[B14] Gitelson A. A. Kaufman Y. J. Merzlyak M. N. (1996). Use of a green channel in remote sensing of global vegetation from EOS-MODIS. Remote Sens. Environ. 58, 289–298. doi: 10.1016/S0034-4257(96)00072-7

[B15] Gitelson A. A. Kaufman Y. J. Stark R. Rundquist D. (2002). Novel algorithms for remote estimation of vegetation fraction. Remote Sens. Environ. 80, 76–87. doi: 10.1016/S0034-4257(01)00289-9

[B16] Gitelson A. Merzlyak M. N. (1994). Spectral Reflectance Changes Associated with Autumn Senescence of Aesculus hippocastanum L. and Acer platanoides L. Leaves. Spectral Features and Relation to Chlorophyll Estimation. J. Plant Physiol. 143, 286–292. doi: 10.1016/S0176-1617(11)81633-0

[B17] Guan S. Fukami K. Matsunaka H. Okami M. Tanaka R. Nakano H.. (2019). Assessing correlation of high-resolution NDVI with fertilizer application level and yield of rice and wheat crops using small UAVs. Remote Sens.-Basel. 11, 112. doi: 10.3390/rs11020112

[B18] Guo Y. Ren G. Zhang K. Li Z. Miao Y. Guo H. (2021). Leaf senescence: progression, regulation, and application. Mol. Hortic. 1, 5. doi: 10.1186/s43897-021-00006-9 37789484 PMC10509828

[B19] Haboudane D. Miller J. R. Pattey E. Zarco-Tejada P. J. Strachan I. B. (2004). Hyperspectral vegetation indices and novel algorithms for predicting green LAI of crop canopies: Modeling and validation in the context of precision agriculture. Remote Sens. Environ. 90, 337–352. doi: 10.1016/j.rse.2003.12.013

[B20] Haboudane D. Tremblay N. Miller J. R. Vigneault P. (2008). Remote estimation of crop chlorophyll content using spectral indices derived from hyperspectral data. IEEE T. Geosci. Remote. 46, 423–437. doi: 10.1109/TGRS.2007.904836

[B21] Hernanda R. A. P. Lee J. Lee H. (2023). Spectroscopy imaging techniques as *in vivo* analytical tools to detect plant traits. Appl. Sci. 13, 10420 . doi: 10.3390/app131810420

[B22] Hunt E. R. Hively W. D. Fujikawa S. Linden D. Daughtry C. S. Mccarty G. (2010). Acquisition of NIR-green-blue digital photographs from unmanned aircraft for crop monitoring. Remote Sens.-Basel. 2, 290–305. doi: 10.3390/rs2010290

[B23] Intergovermental Panel on Climate Change (2022). Climate change 2022 mitigation of climate change. Available online at: https://www.ipcc.ch/report/ar6/wg3.

[B24] Jafarbiglu H. Pourreza A. (2022). A comprehensive review of remote sensing platforms, sensors, and applications in nut crops. Comput. Electron. Agr. 197, 106844. doi: 10.1016/j.compag.2022.106844

[B25] Lao Z. Fu B. Wei Y. Deng T. He W. Yang Y.. (2024). Retrieval of chlorophyll content for vegetation communities under different inundation frequencies using UAV images and field measurements. Ecol. Indic. 158, 111329. doi: 10.1016/j.ecolind.2023.111329

[B26] Li Z. Zhou X. Cheng Q. Fei S. Chen Z. (2023). A machine-learning model based on the fusion of spectral and textural features from UAV multi-sensors to analyse the total nitrogen content in winter wheat. Remote Sens.-Basel. 15, 2152. doi: 10.3390/rs15082152

[B27] Li C. Zhu X. Wei Y. Cao S. Guo X. Yu X.. (2018). Estimating apple tree canopy chlorophyll content based on Sentinel-2A remote sensing imaging. Sci. Rep.-UK. 8, 3756. doi: 10.1038/s41598-018-21963-0 PMC5830534 29491437

[B28] Lu B. He Y. Liu H. H. T. (2018). Mapping vegetation biophysical and biochemical properties using unmanned aerial vehicles-acquired imagery. Int. J. Remote Sens. 39, 5265–5287. doi: 10.1080/01431161.2017.1363441

[B29] Madonsela S. Cho M. A. Naidoo L. Main R. Majozi N. (2023). Exploring the utility of Sentinel-2 for estimating maize chlorophyll content and leaf area index across different growth stages. J. Spat. Sci. 68, 339–351. doi: 10.1080/14498596.2021.2000898

[B30] Maimaitijiang M. Sagan V. Sidike P. Hartling S. Esposito F. (2020). and fritschi, F Soybean yield prediction from UAV using multimodal data fusion and deep learning. B. Remote Sens. Environ. 237, 111599. doi: 10.1016/j.rse.2019.111599

[B31] Main R. Cho M. A. Mathieu R. O Kennedy M. M. Ramoelo A. Koch S. (2011). An investigation into robust spectral indices for leaf chlorophyll estimation. ISPRS J. Photogramm. 66, 751–761. doi: 10.1016/j.isprsjprs.2011.08.001

[B32] Mehmood T. A. B. Ahmed B. C. (2016). The diversity in the applications of partial least squares: an overview. J. Chemometr. 30, 4–17. doi: 10.1002/cem.2762

[B33] Narmilan A. Gonzalez F. Salgadoe A. S. A. Kumarasiri U. W. L. M. Weerasinghe H. A. S. Kulasekara B. R. (2022). Predicting canopy chlorophyll content in sugarcane crops using machine learning algorithms and spectral vegetation indices derived from UAV multispectral imagery. Remote Sens.-Basel. 14, 1140. doi: 10.3390/rs14051140

[B34] Ngo S. H. Kemény S. Deák A. (2003). Performance of the ridge regression method as applied to complex linear and nonlinear models. Chemometr. Intell. Lab. 67, 69–78. doi: 10.1016/S0169-7439(03)00062-5

[B35] Sankaran S. Quirós J. J. Miklas P. N. (2019). Unmanned aerial system and satellite-based high resolution imagery for high-throughput phenotyping in dry bean. Comput. Electron. Agr. 165, 104965. doi: 10.1016/j.compag.2019.104965

[B36] Shanahan J. F. Schepers J. S. Francis D. D. Varvel G. E. Wilhelm W. W. Tringe J. M.. (2001). Use of remote-sensing imagery to estimate corn grain yield. Agron. J. 93, 583–589. doi: 10.2134/agronj2001.933583x

[B37] Sheng Z. Xie S. Q. Pan C. Y. (2020). Probability theory and mathematical statistics (Beijing: Higher Education Press).

[B38] Shu M. Zuo J. Shen M. Yin P. Wang M. Yang X.. (2021). Improving the estimation accuracy of SPAD values for maize leaves by removing UAV hyperspectral image backgrounds. Int. J. Remote Sens. 42, 5862–5881. doi: 10.1080/01431161.2021.1931539

[B39] Tucker C. J. (1979). Red and photographic infrared linear combinations for monitoring vegetation. Remote Sens. Environ. 8, 127–150. doi: 10.1016/0034-4257(79)90013-0

[B40] Wang T. Chandra A. Jung J. Chang A. (2022). UAV remote sensing based estimation of green cover during turfgrass establishment. Comput. Electron. Agr. 194, 106721. doi: 10.1016/j.compag.2022.106721

[B41] Wang B. Chen T. Xu A. (2017). Gaussian process regression with functional covariates and multivariate response. Chemometr. Intell. Lab. 163, 1–6. doi: 10.1016/j.chemolab.2017.02.001

[B42] Wang K. Li W. Deng L. Lyu Q. Zheng Y. Yi S.. (2018). Rapid detection of chlorophyll content and distribution in citrus orchards based on low-altitude remote sensing and bio-sensors. Int. J. Agr. Biol. Eng. 11, 164–169. doi: 10.25165/j.ijabe.20181102.3189

[B43] Xiao B. Li S. Dou S. He H. Fu B. Zhang T.. (2024). Comparison of leaf chlorophyll content retrieval performance of citrus using FOD and CWT methods with field-based full-spectrum hyperspectral reflectance data. Comput. Electron. Agr. 217, 108559. doi: 10.1016/j.compag.2023.108559

[B44] Xu T. Wang F. Xie L. Yao X. Zheng J. Li J.. (2022b). Integrating the textural and spectral information of UAV hyperspectral images for the improved estimation of rice aboveground biomass. Remote Sens.-Basel. 14, 2534. doi: 10.3390/rs14112534

[B45] Xu L. Zhou L. Meng R. Zhao F. Lv Z. Xu B.. (2022a). An improved approach to estimate ratoon rice aboveground biomass by integrating UAV-based spectral, textural and structural features. Precis. Agric. 23, 1276–1301. doi: 10.1007/s11119-022-09884-5

[B46] Yang H. Hu Y. Zheng Z. Qiao Y. Zhang K. Guo T.. (2022). Estimation of potato chlorophyll content from UAV multispectral images with stacking ensemble algorithm. Agron. -Basel. 12, 2318. doi: 10.3390/agronomy12102318

[B47] Yogeshwari M. Thailambal G. (2021). Automatic feature extraction and detection of plant leaf disease using GLCM features and convolutional neural networks. Materials Today: Proc. 81, 530–536. doi: 10.1016/j.matpr.2021.03.700

[B48] Zarco-Tejada P. J. González-Dugo M. V. Fereres E. (2016). Seasonal stability of chlorophyll fluorescence quantified from airborne hyperspectral imagery as an indicator of net photosynthesis in the context of precision agriculture. Remote Sens. Environ. 179, 89–103. doi: 10.1016/j.rse.2016.03.024

[B49] Zhang Z. Zhu L. (2023). A review on unmanned aerial vehicle remote sensing: platforms, sensors, data processing methods, and applications. Drones (Basel). 7, 398. doi: 10.3390/drones7060398

[B50] Zhao C. Zhang Y. Du J. Guo X. Wen W. Gu S.. (2019). Crop phenomics: current status and perspectives. Front. Plant Sci. 10. doi: 10.3389/fpls.2019.00714 PMC6557228 31214228

[B51] Zhou X. Zheng H. B. Xu X. Q. He J. Y. Ge X. K. Yao X.. (2017). Predicting grain yield in rice using multi-temporal vegetation indices from UAV-based multispectral and digital imagery. ISPRS J. Photogramm. 130, 246–255. doi: 10.1016/j.isprsjprs.2017.05.003

